# Body image as risk factor for emotional and behavioral problems among Chinese adolescents

**DOI:** 10.1186/s12889-018-6079-0

**Published:** 2018-10-16

**Authors:** Lingling Ren, Yuanyuan Xu, Xin Guo, Jing Zhang, Hong Wang, Xiaomin Lou, Jianping Liang, Fangbiao Tao

**Affiliations:** 1 School of Public Health, Anhui Medical University, Anhui Provincial Key Laboratory of Population Health & Aristogenics, 81 Meishan Road, Hefei, 230032 Anhui China; 2 Beijing Center for Disease Control and Prevention, Beijing, China; 3 Shenbei District Healthcare Institute for Primary and Middle Schools, Shenyang, China; 4 School of Public Health and Management, Chongqing Medical University, Chongqing, China; 5 School of Public Health, Zhengzhou University, Zhengzhou, China; 6 Guangzhou Primary and Secondary School Health Promotion Center, Guangzhou, China

**Keywords:** Adolescents, Emotional and behavioral problems, Body image, Overweight, Obesity

## Abstract

**Background:**

Being overweight and obesity during adolescence are worldwide public health problems. This study examined the relationship between actual weight, body image, and emotional and behavioral problems among Chinese adolescents.

**Methods:**

A total of 3841 adolescents (age range, 11–16 years) from 5 Chinese cities were included in this cross-sectional study. All of the study participants were asked to complete questionnaires (including demographic features, strengths and difficulties questionnaires, pubertal development scale), and their height and weight were measured at the same time. Body image was measured in two ways: self-perceived weight and body satisfaction. The relationship between weight status and mental health was estimated by multivariate logistic regression for boys and girls.

**Results:**

Our study showed a difference by sex for prevalence of being overweight/obesity and body dissatisfaction among Chinese adolescents. Boys were more likely to be overweight or obese than girls (30.4% vs. 21.5%, *p* < 0.05), but girls were more likely to be dissatisfied with their bodies than boys (41.2% vs. 27.9%, *p* < 0.05). In the logistic regression, body image, not actually being overweight, was significantly associated with a higher risk of emotional and behavioral problems. Compared to perceived normal weight boys, boys who perceived themselves as underweight had an increased likelihood of emotional problems (adjusted odds ratio [OR] = 1.73; 95% confidence interval (CI), 1.16–2.57), conduct problems (OR = 1.73; 95% CI, 1.20–2.50), and total difficulties (OR = 1.50; 95% CI, 1.09–2.05). Compared to body satisfaction, body dissatisfaction was a risk factor for emotional problems (boys: OR = 2.80; 95% CI, 1.84–4.25; girls: OR = 2.18; 95% CI, 1.42–3.36), conduct problems (boys: OR = 1.87, 95% CI, 1.26–2.76; girls: OR = 2.79; 95% CI, 1.46–5.30), hyperactivity problems (boys: OR = 1.67; 95% CI, 1.09–2.55; girls: OR = 2.04; 95% CI, 1.13–3.69), and total difficulties (boys: OR = 2.03; 95% CI, 1.45–2.84; girls: OR = 2.30; 95% CI, 1.46–3.56).

**Conclusions:**

Being overweight and obese during adolescence are very serious public health problems in China. Body image was a more substantial predictor for adolescent emotional and behavioral problems than actually being overweight/obesity.

## Background

Being overweight and obesity in children and adolescents are some of the most pressing public health concerns of the twenty-first century [1]. Recently, obesity was recognized as an illness by the American Medical Association. It has been reported that obesity during childhood or adolescence is associated with the development of numerous diseases, such as asthma, hypertension, dyslipidemia, type 2 diabetes, and fatty liver disease [2–4]. The association between obesity and psychological health among adolescents has also been researched extensively, but the epidemiologic results are inconsistent. Some cross-sectional and prospective studies among children or adolescents supported an association between obesity and mental health problems, including depression, anxiety, low self-esteem, and attention-deficit hyperactivity disorders [5–7]. A study conducted in the United States among 43,297 adolescents reported that obese adolescents have increased odds of reporting poor psychosocial functioning [5]. At the same time, several studies have found no association between obesity and mental health problems when controlling for race, sex, and socioeconomic status [8, 9]. In addition, in some studies, the mental health of boys and girls was affected differently by obesity [10, 11]. In a meta-analysis of 20 studies examining the association between obesity and mental health problems, the association was seen only among girls [10].

Body image refers to the multifaceted psychological experience of embodiment. It encompasses one’s body-related self-perceptions and self-attitudes, including thoughts, beliefs, feelings, and behaviors [12]. Adolescence is a special age with great physical changes; it is also an important period for body image and self-concept development [13]. With the spurt in growth, adolescents are more concerned about their weight status and body shape. With the worldwide adoption of the internet, social networking sites, and mobile apps, adolescents are increasingly exposed to unrealistic and idealized body shapes: a lean, muscular shape for men and a slim shape for women. Great changes in the standards of beauty have occurred alongside the rapid economic development of China. Slimness has become an “ideal” among Chinese adolescents in large cities, especially for girls. Prolonged exposure to unrealistic beauty standards may cause poor body image in adolescents. A high prevalence of poor body image has been reported as a major threat to adolescent health in Western countries [14], and similar results were also obtained in China [15, 16]. Studies have shown the negative psychological effects of poor body image, including anxiety, depression, and low self-esteem [17–20]. Other studies have reported that body image, not actual weight, affected mental health [21–24]. Yu et al. found that perceived overweight adolescents were more likely to experience depressive and anxiety symptoms than perceived normal weight adolescents; no significant association was found between actual weight and symptoms of depression and anxiety [23].

This study aimed to evaluate the associations between emotional and behavioral problems and actual weight, body image among Chinese adolescents. We hypothesized that body image was a more suitable predictor of emotional and behavioral problems than actual weight among Chinese adolescents.

## Materials and methods

### Sample

A total of 4200 adolescents in grades 7 and 8 were selected from 10 middle schools in 5 Chinese cities (Beijing, Shenyang, Zhengzhou, Chongqing, and Guangzhou) by purposive sampling (no less than 200 students of each sex were selected from each school). Written consent from participants and their parents were obtained before investigation. All of the participants were asked to complete questionnaires and measure their height and weight between October 2014 and May 2015. In total, 4087 questionnaires were recovered. Adolescents with missing data, on the questionnaire, for weight, or for height, were excluded from this study. Finally, 3841 adolescents from 11 to 16 years of age were included in this study.

This study was approved by the Ethics Committee of Anhui Medical University. Written consent from all participants and parents were obtained before investigation.

### Measures

#### Actual weight status

Height and weight were measured with calibrated instruments while participants were barefoot and wearing light clothes. Body mass index (BMI) was calculated with the ratio between weight (kg) and the square of height (m^2^). Actual weight status was categorized as underweight, normal weight, or overweight/obesity according to BMI. Overweight and obesity were defined according to the BMI standards put forward by the Working Group on Obesity of China [25]. Underweight was defined according to the BMI cutoff declared by the National Health and Family Planning Commission of China [26].

#### Body image

Body image was measured in two ways: self-perceived weight and body satisfaction. Self-perceived weight status was defined by the question: “What do you think about your own body weight?” There were five options that ranged from “very underweight” to “very overweight”. In the data analysis, the “very underweight” and “somewhat underweight” options were categorized as “underweight”; “somewhat overweight” and “very overweight” were categorized as “overweight”, and “about the right weight” was categorized as “normal weight”. Thus, there were three categories for self-perceived weight status: perceived underweight, perceived normal weight and perceived overweight.

Body satisfaction status was assessed through the question “Are you satisfied with your body weight?” The answers included 5 options from “very dissatisfied” to “very satisfied”. In the data analysis, the options “very dissatisfied” and “somewhat dissatisfied” were combined into “dissatisfaction”, and “somewhat satisfied” and “very satisfied” were combined into “satisfaction”. Thus, there were three categories for body satisfaction status: dissatisfaction, neither, and satisfaction.

#### Emotional and behavioral problems

Emotional and behavioral problems were assessed by the strengths and difficulties questionnaires (SDQs). There were three versions of the SDQ: a parent form, a teacher form, and a self-report form [27]. In this study, the self-report form was used. The Chinese version of the self-report SDQ has been validated in China. Overall Cronbach’s alpha coefficient was 0.81 [28]. The SDQ contained 25 items and generated scores for 5 subscales: emotional symptoms, conduct problems, hyperactivity problems, peer problems, and prosocial behavior. Response options for each item are “not true,” “somewhat true,” or “certainly true,” which scored 0, 1, and 2, respectively. It should be noted that 5 positively worded questions were reverse scored. The score for every subscale was generated by summing the scores of the 5 corresponding items. The total difficulties score was the sum score of emotional symptoms, conduct problems, hyperactivity problems, and peer problems. A higher score indicated less difficulty for prosocial behavior but more difficulties for the other subscales. Cutoff scores recommended by Du et al. for Chinese adolescents were used to identify “case” participants (emotional symptoms > 5, conduct problems > 4, hyperactivity problems > 6, peer problems > 5, prosocial behavior < 5, and total difficulties > 17) [29].

#### Covariates

Socioeconomic and demographic factors including age, parents’ education level, number of children in family, household income level, and parents smoking were recorded. The parents’ education level was categorized as below high school or high school graduate and above. Household income level was divided into 3 categories: below moderate, moderate, and above moderate.

In this study, the pubertal development stage was also selected as a covariate because it had been reported that early puberty was related to emotional and behavioral problems among adolescents [30]. The “pubertal development scale” (PDS) developed by Peterson was used to measure the pubertal development stage [31]. The Chinese version of self-report PDS had been evaluated among Chinese children [32]. The questionnaire included 5 items: growth spurt, skin changes, pubic hair growth for both sexes, breast development and menarche onset for girls, facial hair and voice change for boys. The answers for each item (except for menarche) included 4 options scored from 1 (no development) to 4 (development is completed). Menarche was scored as 4 for “menarche” and 1 for “no menarche.” The PDS scores for every adolescent were summarized, averaged, and standardized within each sex and age (in years). Pubertal status was categorized as early, on-time, or late maturation, according to methods previously outlined [33].

#### Analyses

Statistical analysis was conducted with SPSS 16.0. Data were analyzed descriptively to show the demographic factors of the samples. The prevalence of actual weight, perceived weight, and body dissatisfaction of the study participants were tested by a chi-squared test. The association between weight status and mental health was estimated with multivariate logistic regression for boys and girls. In the logistic regression, weight status was an independent variable, emotional and behavior problems were dependent variables, and socioeconomic characteristics and pubertal development stage were confounding factors. We performed 2 types of regression. First, the associations between emotional and behavior problems and actual weight status, self-perceived weight status, and body satisfaction status were examined respectively, after adjusting for confounding factors. Second, the associations between emotional and behavior problems and actual weight status, self-perceived weight status, and body satisfaction status were examined simultaneously, after adjusting for confounding factors. The adjusted odds ratio (OR) and their 95% confidence intervals were reported. A *p* value of < 0.05 was considered to be statistically significant.

## Results

General characteristics of the sample are summarized in Table 1. A total of 3841 adolescents were included in this study, with an age range of 11 to 16 years. The sample was almost equally composed of boys (*n* = 1927, 50.2%) and girls (*n* = 1914, 49.8%). The mean age of participants was 13.4 (SD = 0.8). Children were divided by age into 3 groups: under 13 years (*n* = 1267, 33.0%), 13–14 years (*n* = 1754, 45.6%), and more than 14 years (*n* = 820, 21.3%). A little of more than half of the parents had an education level of high school graduate and above (father: *n* = 2195, 57.1%; mother: *n* = 2098, 54.6%). Household income level was divided into 3 categories: below moderate (*n* = 273, 7.1%), moderate (*n* = 3061, 79.7%), and above moderate (*n* = 507, 13.2%). Most participants came from a family with only one child (*n* = 2403, 62.6%). Most participants belonged to the on-time maturation group (*n* = 2642, 68.8%). For emotional and behavior problems, 11.7% reported emotional symptoms, 9.6% conduct problems, 8.1% hyperactivity problems, 13.4% peer problems, 8.7% prosocial behavior, and 16.2% total difficulties.

**Table 1:** General characteristics of the samples

	Boys (*N* = 1927)	Girls (*N* = 1914)	Total (*N* = 3841)
	N or mean (% or SD)	N or mean (% or SD)	N or mean (% or SD)
Age	13.4 (0.7)	13.3 (0.8)	13.4 (0.8)
< 13	586 (30.4)	681 (35.6)	1267 (33.0)
13–14	922 (47.8)	832 (43.5)	1754 (45.7)
≥ 14	419 (21.9)	401 (20.9)	820 (21.3)
Father’s education			
< high school	815 (42.3)	831 (43.4)	1646 (42.9)
≥ high school	1112 (57.7)	1083 (56.6)	2195 (57.1)
Mother’s education			
< high school	905 (47.0)	838 (43.8)	1743 (45.4)
≥ high school	1022 (53.0)	1076 (56.2)	2098 (54.6)
House hold income level			
Under moderate	175 (9.1)	98 (5.1)	273 (7.1)
Moderate	1461 (75.8)	1600 (83.6)	3061 (79.7)
Over moderate	291 (15.1)	216 (11.3)	507 (13.2)
One child			
Yes	1256 (65.2)	1147 (59.9)	2403 (62.6)
No	671 (34.8)	767 (40.1)	1438 (37.4)
Pubertal development stage			
Early	265 (13.8)	315 (16.5)	580 (15.1)
On time	1364 (70.8)	1278 (66.8)	2642 (68.8)
Late	298 (15.5)	321 (16.8)	619 (16.1)
Parents smoking			
Yes	1060 (55.0)	1078 (56.3)	2138 (55.7)
No	867 (45.0)	836 (43.7)	1703 (44.3)
Emotional and behavioral problems*			
Emotional symptoms	204 (10.6)	244 (12.7)	448 (11.7)
Conduct problems	241 (12.5)	128 (6.7)	369 (9.6)
Hyperactivity problems	179 (9.3)	131 (6.8)	310 (8.1)
Peer problems	268 (13.9)	248 (13.0)	516 (13.4)
Prosocial behaviour	240 (12.5)	96 (5.0)	336 (8.7)
Total difficulties	357 (18.5)	267 (13.9)	624 (16.2)

*the proportion of “case” in every subscale of emotional and behavioral problems

Table 2 shows the actual weight, self-perceived weight, and body dissatisfaction data. For actual weight, there was a significant difference in the prevalence of being overweight or obesity according to sex (*χ*^*2*^ *=* 40.0, *p* < 0.001), age (*χ*^*2*^ *=* 11.7, *p* < 0.05), one-child (*χ*^*2*^ *=* 22.60, *p* < 0.001), household income level (*χ*^*2*^ *=* 8.4, *p* < 0.05), parents smoking (*χ*^*2*^ *=* 5.4, *p* < 0.05), and pubertal development stage (*χ*^*2*^ *=* 15.7, *p* < 0.001). The prevalence of underweight among girls was significantly higher than among boys (*χ*^*2*^ *=* 26.9, *p* < 0.001), but boys were more likely than girls to perceive themselves as underweight (*χ*^*2*^ *=* 45.3, *p* < 0.001). Compared to boys, girls are more likely to be dissatisfied with their body weight (*χ*^*2*^ *=* 74.4, *p* < 0.01). The prevalence of body dissatisfaction increased with age (*χ*^*2*^ *=* 6.2, *p* < 0.05). Adolescents from low-income households were more likely to be dissatisfied with their body than those from moderate and over-moderate income households (*χ*^*2*^ *=* 6.5, *p* < 0.05). Pubertal development stage had a significant effect on both actual weight and body image. Adolescents with early puberty were the most likely to perceive themselves as being overweight (*χ*^*2*^ *=* 20.0, *p* < 0.001). Adolescents coming from one-child families were more likely to be overweight or obese than those from multiple children families (*χ*^*2*^ *=* 22.6, *p* < 0.01). The prevalence of overweight and body dissatisfaction was higher among adolescents with parents who smoked than among those with parents who did not smoke (overweight: *χ*^*2*^ *=* 5.4, *p* < 0.05; body dissatisfaction: *χ*^*2*^ *=* 9.6, *p* < 0.05).

**Table 2:** Distribution of actual weight, self-perceived weight, and body satisfaction status in study participants

Demographic factors		Prevalence (%)						
		Actual weight		Self-perceived weight		Body satisfaction		
		Underweight	Overweight/obesity	Underweight	Overweight	Satisfaction	Neither	Dissatisfaction
Sex	Male	3.6	30.4	28.6	28.5	35.7	36.4	27.9
	Female	7.4	21.5	19.3	29.7	26.7	32.1	41.2
	*χ*^*2*^(*p*)	26.9 (*p* < 0.001)	40.0 (*p* < 0.001)	45.3(*p* < 0.001)	0.7 (*p* > 0.05)	36.2 (*p* < 0.001)	7.7 (*p* < 0.05)	74.4 (*p* < 0.001)
Age	< 13	4.3	28.9	22.3	29.2	34.9	32.9	32.2
	13–14	5.7	25.6	24.5	29.0	29.7	35.5	34.8
	> 14	7.1	22.2	25.4	28.9	28.8	33.7	37.5
	*χ*^*2*^(*p*)	7.8 (*p* < 0.05)	11.7 (*p* < 0.05)	3.1 (*p* > 0.05)	0.02(*p* > 0.05)	12.8 (*p* < 0.05)	2.5 (*p* > 0.05)	6.2 (*p* < 0.05)
One child	Yes	5.3	28.6	23.5	29.7	31.7	34.6	33.7
	No	5.8	21.6	24.9	28.0	30.0	33.9	36.1
	*χ*^*2*^(*p*)	0.4(*p* > 0.05)	22.6 (*p* < 0.001)	1.0 (*p* > 0.05)	1.8 (*p* > 0.05)	1.2 (*p* > 0.05)	0.2 (*p* > 0.05)	2.2 (*p* > 0.05)
Household income level	Under moderate	7.4	27.0	27.4	31.1	28.6	29.7	41.7
	Moderate	5.6	24.6	24.1	28.2	30.9	35.1	34.0
	Over moderate	4.0	25.7	21.7	32.5	34.2	31.8	34.0
	*χ*^*2*^(*p*)	4.0 (*p* > 0.05)	8.4 (*p* < 0.05)	3.1 (*p* > 0.05)	4.3 (*p* > 0.05)	3.0 (*p* > 0.05)	4.7(*p* > 0.05)	6.5(*p* < 0.05)
Parents smoking	Yes	4.9	27.4	23.6	30.6	29.3	34.0	36.7
	No	6.3	24.1	24.7	27.2	33.6	34.5	31.9
	*χ*^*2*^(*p*)	3.7 (*p* > 0.05)	5.4 (*p* < 0.05)	0.7 (*p* > 0.05)	5.5 (*p* < 0.05)	8.2 (*p* < 0.05)	0.1 (*p* > 0.05)	9.6 (*p* < 0.05)
Pubertal development stage	Early	3.70	30.5	17.8	35.4	26.7	35.1	38.2
	On time	4.89	26.1	23.2	28.8	32.2	33.6	32.2
	Late	10.9	20.5	34.0	23.4	31.7	36.2	34.2
	*χ*^*2*^(*p*)	38.4 (*p* < 0.001)	15.7 (*p* < 0.001)	45.6(*p* < 0.001)	20.0(*p* < 0.001)	7.1 (*p* < 0.05)	1.6 (*p* > 0.05)	5.3 (*p* > 0.05)

The adjusted odds of mental health problems, after controlling for age, parents’ education level, household income level, pubertal development stage, and parents smoking, are reported in Table 3 for girls and Table 4 for boys.

**Table 3:** Association between actual weight, self-perceived weight, body dissatisfaction, and mental health for girls

Weight status	OR (95% CI)					
	Emotional	Conduct	Hyperactivity	Peer problems	Prosocial	Total difficulties
Model1: Actual weight						
Normal weight	Ref.	Ref.	Ref.	Ref.	Ref.	Ref.
Underweight	1.73 (0.88–3.41)	1.18 (0.76–1.84)	0.78 (0.23–2.59)	1.35 (0.64–2.86)	1.86 (0.40–3.48)	0.84 (0.37–1.91)
Overweight/obesity	1.28 (0.91–1.80)	0.22 (0.03–1.66)	0.96 (0.60–1.52)	1.59 (1.15–2.19)**	1.46 (0.90–2.38)	1.49 (1.09–2.04)*
Model 2: Perceived weight						
Perceived normal weight	Ref.	Ref.	Ref.	Ref.	Ref.	Ref.
Perceived underweight	1.10 (0.73–1.65)	0.38 (0.19–0.79)*	0.86 (0.49–1.53)	1.30 (0.95–1.79)	1.28 (0.72–2.28)	0.72 (0.47–1.11)
Perceived overweight	1.58 (1.15–2.18)**	1.49 (0.99–2.23)	1.28 (0.84–1.95)	0.96 (0.64–1.43)	1.37 (0.84–2.22)	1.74 (1.29–2.35)**
Model 3: Body satisfaction						
Satisfaction	Ref.	Ref.	Ref.	Ref.	Ref.	Ref.
Neither	0.96 (0.61–1.51)	1.45 (0.74–2.83)	1.15 (0.62–2.12)	0.99 (0.67–1.46)	1.61 (0.86–3.02)	1.44 (0.92–2.24)
Dissatisfaction	2.26 (1.52–3.35)**	3.25 (1.79–5.92)**	2.06 (1.19–3.56)*	1.12 (0.78–1.61)	1.79 (0.98–3.27)	2.78 (1.86–4.16)**
Model 4: Actual weight + perceived weight + body satisfaction weight						
Normal weight	Ref.	Ref.	Ref.	Ref.	Ref.	Ref.
Underweight	2.04 (0.96–4.34)	0.53 (0.07–4.32)	1.02 (0.28–3.68)	1.52 (0.68–3.40)	1.08 (0.34–3.45)	1.27 (0.52–3.11)
Overweigh/obesity	0.89 (0.60–1.34)	0.68 (0.41–1.15)	0.73 (0.43–1.26)	1.54 (1.03–2.29)*	1.33 (0.73–2.44)	0.97 (0.67–1.42)
Perceived normal weight	Ref.	Ref.	Ref.	Ref.	Ref.	Ref.
Perceived underweight	0.96 (0.61–1.52)	0.40 (0.18–0.90)*	0.84 (0.45–1.58)	0.90 (0.58–1.39)	1.26 (0.67–2.38)	0.72 (0.45–1.17)
Perceived overweight	1.20 (0.81–1.76)	1.30 (0.80–2.12)	1.14 (0.69–1.89)	1.08 (0.72–1.61)	1.00 (0.55–1.83)	1.36 (0.94–1.96)
Satisfaction	Ref.	Ref.	Ref.	Ref.	Ref.	Ref.
Neither	0.95 (0.60–1.51)	1.33 (0.67–2.16)	1.14 (0.62–2.11)	0.93 (0.62–1.37)	1.61 (0.85–3.04)	1.33 (0.85–2.08)
Dissatisfaction	2.18 (1.42–3.36)**	2.79 (1.46–5.30)**	2.04 (1.13–3.69)*	0.93 (0.62–1.40)	1.71 (0.89–3.29)	2.30 (1.48–3.56)**

All of the models were controlled for age, parents’ education level, household income level, pubertal development stage, and parents smoking

*p < 0.05; ** *p* < 0.01

**Table 4:** Association between actual weight, self-perceived weight, body dissatisfaction, and mental health for boys

Weight status	OR (95% CI)					
	Emotional	Conduct	Hyperactivity	Peer problems	Prosocial	Total difficulties
Model 1: Actual weight						
Normal weight	Ref.	Ref.	Ref.	Ref.	Ref.	Ref.
Underweight	0.74 (0.39–1.43)	0.44 (0.21–0.92)*	1.10 (0.61–1.99)	1.02 (0.59–1.79)	0.71 (0.38–1.33)	0.57 (0.33–0.99)*
Overweight/obesity	1.18 (0.85–1.64)	1.08 (0.79–1.47)	0.92 (0.64–1.33)	1.70 (1.27–2.27)**	0.94 (0.68–1.30)	1.11 (0.85–1.44)
Model 2: Perceived weight						
Perceived normal weight	Ref.	Ref.	Ref.	Ref.	Ref.	Ref.
Perceived underweight	1.59 (1.10–2.30)*	1.49 (1.05–2.11)*	1.16 (0.79–1.69)	1.15 (0.82–1.62)	0.89 (0.63–1.27)	1.34 (1.01–1.80)*
Perceived overweight	1.61 (1.11–2.34)*	1.56 (1.11–2.21)*	1.05 (0.71–1.57)	1.80 (1.31–2.48)**	1.02 (0.72–1.44)	1.45 (1.08–1.95)*
Model 3: Body satisfaction						
Satisfaction	Ref.	Ref.	Ref.	Ref.	Ref.	Ref.
Neither	1.31 (0.87–1.97)	1.39 (0.97–1.98)	0.96 (0.64–1.43)	0.91 (0.65–1.28)	1.39 (0.99–1.97)	1.34 (0.99–1.82)
Dissatisfaction	2.69 (1.82–3.96)**	1.86 (1.30–2.67)**	1.52 (1.03–2.25)*	1.52 (1.09–2.12)*	1.12 (0.77–1.65)	1.99 (1.46–2.71)**
Model 4: Actual weight + perceived weight + body satisfaction weight						
Normal weight	Ref.	Ref.	Ref.	Ref.	Ref.	Ref.
Underweight	0.51 (0.26–1.02)	0.32 (0.15–0.68)**	0.99 (0.52–1.88)	0.93 (0.52–1.68)	0.68 (0.34–1.35)	0.44 (0.25–0.78)
Overweight/obesity	0.97 (0.59–1.58)	0.90 (0.57–1.41)	0.79 (0.47–1.32)	1.37 (0.90–2.08)	0.83 (0.53–1.30)	0.94 (0.64–1.39)
Perceived normal weight	Ref.	Ref.	Ref.	Ref.	Ref.	Ref.
Perceived underweight	1.73 (1.16–2.57)**	1.73 (1.20–2.50)**	1.09 (0.72–1.65)	1.22 (0.84–1.77)	0.93 (0.64–1.36)	1.50 (1.09–2.05)*
Perceived overweight	1.15 (0.70–1.90)	1.37 (0.86–2.17)	1.01 (0.60–1.71)	1.35 (0.881–2.07)	1.13 (0.72–1.78)	1.17 (0.79–1.74)
Satisfaction	Ref.	Ref.	Ref.	Ref.	Ref.	Ref.
Neither	1.33 (0.88–2.01)	1.40 (0.98–2.01)	0.99 (0.66–1.48)	0.85 (0.60–1.19)	1.40 (0.98–1.98)	1.36 (1.00–1.85)
Dissatisfaction	2.80 (1.84–4.25)**	1.87 (1.26–2.76)**	1.67 (1.09–2.55)*	1.22 (0.85–1.76)	1.14 (0.75–1.72)	2.03 (1.45–2.84)**

All of the models were controlled for age, parents’ education level, household income level, pubertal development stage, and parents smoking

*p < 0.05; **p < 0.01

Model 1 showed the association between actual weight and mental health. Actual overweight and obesity increased the risk of peer problems for both sexes (girls: OR = 1.59; 95% CI, 1.15–2.19; boys: OR = 1.70; 95% CI, 1.27–2.27). Actual overweight and obesity was also a risk factor of total difficulties for girls (OR = 1.49; 95% CI = 1.09–2.04). However, actually being underweight decreased the risk of conduct problems and total difficulties for boys (conduct problems: OR = 0.44; 95% CI, 0.21–0.92; total difficulties: OR = 0.57; 95% CI = 0.33–0.99).

Model 2 showed the association between self-perceived weight and mental health. It was found that girls who perceived themselves to be overweight reported 1.58-fold (95% CI, 1.15–2.18) emotional symptoms and 1.74-fold (95% CI, 1.29–2.35) total difficulties than girls who perceived themselves as normal weight. Perceived underweight was a protective factor for conduct problems (OR = 0.38; 95% CI, 0.19–0.79) for girls. For boys, both perceived underweight and perceived overweight were positively associated with most emotional and behavioral problems, including emotional symptoms (perceived underweight: OR = 1.59, 95% CI, 1.10–2.30; perceived overweight: OR = 1.61; 95% CI, 1.11–2.34), conduct problems (perceived underweight: OR = 1.49; 95% CI, 1.05–2.11; perceived overweight: OR = 1.56; 95% CI, 1.11–2.21), peer problems (perceived overweight: OR = 1.80; 95% CI, 1.31–2.48), and total difficulties (perceived underweight: OR = 1.34; 95% CI, 1.01–1.80; perceived overweight: OR = 1.45; 95% CI, 1.08–1.95).

The results of model 3 with body dissatisfaction as an independent variable showed that body dissatisfaction was significantly associated with emotional symptoms (girls: OR = 2.26; 95% CI, 1.52–3.35; boys: OR = 2.69; 95% CI, 1.82–3.96), conduct problems (girls: OR = 3.25; 95% CI, 1.79–5.92; boys: OR = 1.86; 95% CI, 1.30–2.67), hyperactivity problems (girls: OR = 2.06; 95% CI, 1.19–3.56; boys: OR = 1.52, 95% CI, 1.03–2.25), peer problems (boys: OR = 1.52; 95% CI, 1.09–2.12), and total difficulties (girls: OR = 2.78; 95% CI, 1.86–4.16; boys: OR = 1.99; 95% CI, 1.46–2.71) for both sexes.

Model 4 included actual weight, self-perceived weight, and body dissatisfaction as predictors simultaneously. In this model, the association between mental health problems and actual overweight or obesity was not seen except for an increased risk of peer problems among girls (OR = 1.54; 95% CI, 1.03–2.29). Compared to boys who perceived themselves as being normal weight, boys who perceived themselves as being underweight had greater odds of emotional symptoms (OR = 1.73; 95% CI, 1.16–2.57), conduct problems (OR = 1.73; 95% CI, 1.20–2.50), and total difficulties (OR = 1.50; 95% CI, 1.09–2.05). Body dissatisfaction was still a risk factor of most emotional and behavioral problems (emotional, conduct, hyperactivity problems, and total difficulties) for both sexes.

## Discussion

This study found that adolescent boys were more likely than girls to be actually overweight and yet they perceive themselves as underweight. Girls were more likely than boys to be dissatisfied with their body weight. These results are consistent with previous studies conducted in Western countries and China [16, 19, 23, 34, 35]. Perceived weight and body satisfaction were important aspects of body image that can be influenced by media and the predominant culture. Influenced by traditional attitudes, some Chinese parents believed that a slightly higher body mass for boys was considered a symbol of physical health and cute, especially in some rural areas. This may be the reason for the higher prevalence of being overweight or obesity among boys. However, in some urban areas, a slim physique was more popular, especially for girls, even if the ideal shape was unrealistic. Compared to boys, girls were more concerned about their body weight and were more likely to be influenced by peers, mass media, parents, and other socio-cultural factors that promote thinness for girls, even if they were normal weight. This may be the reason for the higher prevalence of body dissatisfaction among girls.

The prevalence of being overweight or obesity was higher in this study than in previous studies [36]. This result suggests that being overweight or obesity in Chinese adolescents have become a very serious public health problem and must be taken into account. More than half of the adolescents were from one-child families in this study, which may be one reason for the higher prevalence of being overweight or obesity. Without siblings, the child may receive more attention and family resources, which includes more energy-rich foods and less housework.

We examined the association between mental health and weight status, including actual weight, self-perceived weight, and body dissatisfaction simultaneously. When all factors, including actual weight, self-perceived weight, and body dissatisfaction, were included in a regression model, there was no association between actual overweight or obesity and mental health, except for an increased risk of peer problems among girls. These results suggest that actual overweight may have less of influence on adolescent mental health than perceived weight. Boys and girls were affected differently by weight perception. Perceived underweight increased the risk of emotional problems, conduct problems, and total difficulties in boys, but it decreased risk of conduct problems in girls. Previous studies obtained similar results for boys [37, 38]. These results may be explained by the fact that girls want to be thinner but boys want to be stronger and more muscular.

A previous study reported that adolescents with body dissatisfaction disclosed more psychosocial health problems [39], and our results support this finding. In this study, it was found that body dissatisfaction significantly affected mental health in both sexes. Our results, together with other similar studies [19, 23, 24, 37, 40], confirmed that body image (including perceived weight and body satisfaction) was a more substantial predictor of adolescent emotional and behavioral problems than actual overweight or obesity. Few previous studies simultaneously included actual weight, perceived weight, and body satisfaction. A study that examined the association between depression and actual weight, perceived weight, and body dissatisfaction reported that self-perceived weight, not actual obesity or body dissatisfaction, was a risk factor for depression among adolescents [28], which was different from our outcomes. This difference may result from the difference in sample ethnicity between the two studies. In addition, the previous study only examined the relationship between weight status and depression; our study, however, examined the relationship between weight status and emotional and behavioral problems.

### Strengths and limitations

The strengths of this study are the large sample size and high response rate. However, our study also had some limitations. First, this study was cross-sectional and could not detect relationships as well as a longitudinal study. Second, we did not conduct a subsample comparison analysis. Finally, this study did not consider the effects of resident address or parental mental health. In the future, we will examine the association between body image and emotional and behavioral problems with a longitudinal study that includes more socio-demographic factors and subsamples.

## Conclusion

Our results confirmed that adolescent overweight/obesity is a very serious public health problem, and the prevalence of single-child households may be one reason for the high prevalence of being overweight and obesity in China. Body image (including perceived weight and body satisfaction) was a more substantial predictor of adolescent emotional and behavioral problems than actual overweight or obesity. Adolescents in China need more guidance to distinguish between healthy and unrealistic weight standards.

## Abbreviations

BMI: Body mass index
CI: Confidence interval
OR: Odds ratio
PDS: Pubertal development scales
SD: Standard deviation
SDQ: Strengths and difficulties questionnaires
